# Improving Ammonia Detecting Performance of Polyaniline Decorated rGO Composite Membrane with GO Doping

**DOI:** 10.3390/ma14112829

**Published:** 2021-05-25

**Authors:** Yubin Yuan, Haiyang Wu, Xiangrui Bu, Qiang Wu, Xuming Wang, Chuanyu Han, Xin Li, Xiaoli Wang, Weihua Liu

**Affiliations:** 1 School of Microelectronics, School of Electronics and Information Engineering, Xi’an Jiaotong University, Xi’an 710049, China; yuan8262xy@stu.xjtu.edu.cn (Y.Y.); wuhaiyang@stu.xjtu.edu.cn (H.W.); bxr1212@stu.xjtu.edu.cn (X.B.); w18801757@163.com (Q.W.); xmwang_zw@126.com (X.W.); hanchuanyu@mail.xjtu.edu.cn (C.H.); lx@mail.xjtu.edu.cn (X.L.); xlwang@mail.xjtu.edu.cn (X.W.); 2 The Key Lab of Micro-nano Electronics and System Integration of Xi’an City, Xi’an 710049, China; 3 Guangdong Shunde Xi’an Jiaotong University Academy, Xi’an Jiaotong University, NO.3 Deshengdong Road, Daliang, Shunde District, Foshan 528300, China; 4 School of Science, Xi’an Jiaotong University, Xi’an 710049, China; 5 Research Institute of Xi’an Jiaotong University, Hangzhou 311215, China

**Keywords:** gas sensor, nanocomposites, reduced graphene oxide, graphene oxide, PA-PANI, protic acid doping, composite membrane

## Abstract

Gas-sensing performance of graphene-based material has been investigated widely in recent years. Polyaniline (PANI) has been reported as an effective method to improve ammonia gas sensors’ response. A gas sensor based on a composite of rGO film and protic acid doped polyaniline (PA-PANI) with GO doping is reported in this work. GO mainly provides NH_3_ adsorption sites, and PA-PANI is responsible for charge transfer during the gas-sensing response process. The experimental results indicate that the NH_3_ gas response of rGO is enhanced significantly by decorating with PA-PANI. Moreover, a small amount of GO mixed with PA-PANI is beneficial to increase the gas response, which showed an improvement of 262.5% at 25 ppm comparing to no GO mixing in PA-PANI.

## 1. Introduction

Since the 21st century, information technology has become a prominent feature of human society’s development and progress. In order to realize the interconnection of everything, various sensors with different layout types in production and life are needed as information acquisition terminals. Traditionally, gas sensors have been more applied in many aspects of industrial, medical, and environmental protection. With the development of Internet of Things (IoT) technology, gas sensors will penetrate many scenes of life: food production and safety [1,2], human health status detection [3,4], and home environment [5,6], etc. Due to the requirements of high accuracy, miniaturization, and low power consumption for gas sensors in the IoT application, micro gas sensors with integration convenience demonstrated prominent potential for further study [7,8]. Compared to traditional metal oxide gas sensors, sensors based on 2-dimensional semiconductor materials do not have to rely on a high-temperature working environment, which shows the prospect of low-energy and application in portable Internet of Things devices. As the first 2D semiconductor material prepared in the laboratory, graphene is a planar allotrope of carbon. All carbon atoms in graphene are connected in the form of covalent bonds in a single plane [9]. This is because, in graphene, the 2s orbitals of carbon atoms form sp_2_ hybrid orbitals with 2p_x_ and 2p_y_ orbitals. Thus, carbon atoms can bond to each other tightly through σ-bands, which have the electrons localization property to generate the morphology of plane connecting carbon atoms [9,10]. Therefore, graphene has large specific surface areas for gas molecule adsorption. Besides, 2p_z_ electrons form the weakly bounded π-bands to increase electrons transport on the surface of graphene, leading to excellent electrical properties and low current noise. These basic characteristics indicate that graphene-based two-dimensional materials show great potential in gas-sensing applications [11]. However, because of lacking dangling bonds on the surface, molecular adsorption is usually formed through Van der Waals interaction, which leads to limited gas adsorption capacity and selectivity of intrinsic graphene [12]. Thus, researchers tended to make some decorations on graphene to get a composite film. Decoration options like metal nanoparticles such as Pd [13], Pt [14], and Ag [15] and metal oxides like SnO_2_ [16], PdO [17], and Cu_2_O [18], etc. all presented the enhancement of gas-sensing performance, especially selectivity of graphene-based gas sensor.

Another possible choice is synthesizing functionalized graphene, like graphene oxide(GO) and reduced graphene oxide(rGO) [19,20,21]. Compared to intrinsic graphene, GO and rGO have a certain amount of groups such as carboxyl, hydroxyl, and epoxy on their surface [22]. As these functional groups could provide adsorption sites for gas molecules, GO and rGO have demonstrated their great potential for gas detecting. Recently, we have synthesized an oxygen plasma-treated CVD graphene/intrinsic graphene composite membrane for gas sensing [23]. The composite film reported in that work exhibited a fast response and recovery process, combining with high sensitivity. However, note that with many functional groups on its surface, GO inevitably shows poor conductivity as a characteristic of oxidation state [24,25]. In most cases, poor conductivity usually leads to high current noise in gas detecting. Moreover, after exposure in gas environments for a long time, dangling bonds on the surface of GO would be partially removed, which changes the character of GO and decreases the long-term stability [26]. When treating GO with reducing agents, oxygen-containing groups are removed extensively, which can increase conductance and long-term stability [27]. Further, if the time of reducing action is controlled precisely, we can get partially reduced GO, which reserves a fraction of surface groups. Partially reduced GO balances the property of electric conductivity and gas molecular adsorption capability.

Meanwhile, conductive polymers, such as polyaniline (PANI), have excellent gas sensitivity, acceptable conductivity, and excellent stability [28]. Polyaniline has been widely investigated and used because of its simple synthesis process, good chemical stability, and environmental friendliness. When treating with protic acid, PANI could gain proton from protic acid and form N^+^-H bonds to increase charge transport property. Thus, PANI has H^+^ transport on its molecular link, which usually demonstrates p-type doping [29,30]. Based on the conductive mechanism mentioned above, gas molecules, especially reducing gases, could deliver electrons to PANI and form de-doping, which gives another possible gas detection choice. Inspired by those basic theories, in this work, we use rGO instead of graphene as a conductive channel, and a layer of protic acid doped polyaniline (PA-PANI) is loaded on its top surface for ammonia detection. The composite film shows good ammonia detection performance. More importantly, when a small amount of GO is mixed in PA-PANI, the response is significantly improved by 262.5% to 25 ppm ammonia. The double-layer composite sensitive membrane also delivered the potential of gate tunable gas sensitivity for further study.

## 2. Materials and Methods

### 2.1. Sensor Fabrication

**Figure 1:**
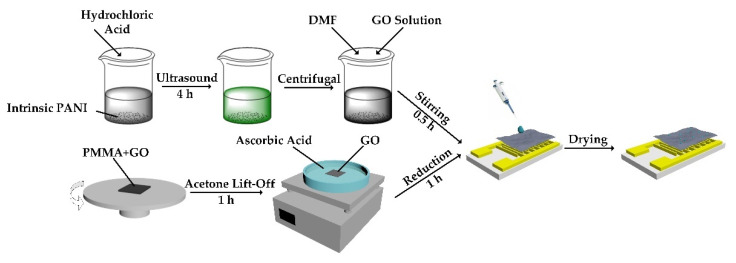
The schematic of the device fabrication process.

The schematic of the device fabrication process is depicted in Figure 1. The preparation method of PA-PANI is shown as follows. Firstly, 0.5 g polyaniline (98% purified, purchased from Cool Chemical Technology Co., Ltd., Beijing, China) was mixed with 50 mL hydrochloric acid (36–38%, purchased from China National Pharmaceutical Group Co., Ltd., Shanghai, China) after diluting the hydrochloric acid to pH = 1. The mixed suspension was treated with ultrasound to synthesize PA-PANI. Secondly, the synthesized suspension was centrifuged with DI water until the washing solution reached pH = 7, followed by centrifuged with ethanol twice. Thirdly, 0.15 g PA-PANI was dissolved in 30 mL N, *N*-Dimethylformamide (DMF) (≥99.5%, purchased from China National Pharmaceutical Group Co., Ltd., Shanghai, China) to form 5 mg/mL PA-PANI solution, which was mixed with 1.2 mg/mL GO (purchased from XFNANO Materials Technology Co., Ltd., Nanjing, China) dispersion subsequently to get different mass ratios of PA-PANI and GO among 30:0.5, 30:1, 30:1.5, and 30:2. The synthesis of rGO films also proceeded simultaneously. Firstly, the silicon substrate was cut into small cube pieces in order to fit the size of the interdigital electrode (IDE). The IDEs were fabricated on Al_2_O_3_ substrate with a size of 10 mm × 5 mm × 0.635 mm. There were seven pairs of Au electrodes with a width of 200 μm and a spacing of 200 μm on the substrate. Then, polymethyl methacrylate (PMMA, purchased from Micro Chem, Westborough, MA, USA) and GO dispersion were successively spin-coated on the silicon wafer and dried. Secondly, the Si/PMMA/GO structure was immersed in acetone to remove PMMA. After the complete GO film was “lift-off” from the silicon substrate and then transferred into 10 mg/mL L(+)- ascorbic acid (AR, purchased from China National Pharmaceutical Group Co., Ltd., Shanghai, China) solution for reducing treatment. In the end, the mixed PA-PANI and GO solution was drop-coasted onto the rGO surface, which had already been transferred onto the interdigital electrode (IDEs). The sensor’s fabrication was finished after drying. Polyaniline before and after protic acid treating are shown in Figure S1.

### 2.2. Measurement Setup

**Figure 2:**
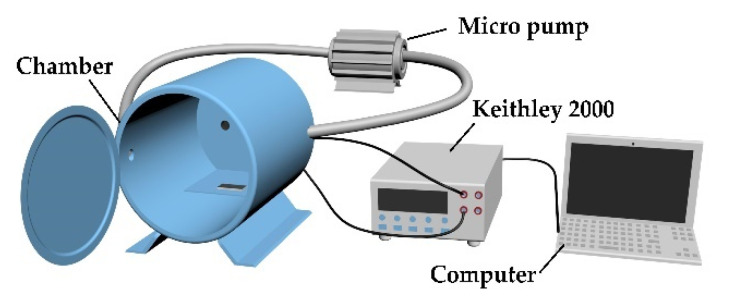
Schematic diagram of the gas-sensing test system.

Figure 2 is a schematic diagram of the gas-sensing test system. The sensor was placed in an acrylic chamber with a volume of 4.67 L. The two ports of the chamber were connected to a pump to make sure the air current circulation in the sealed environment during testing. The concentration of NH_3_ was controlled by the volume of gas injected through a syringe into the testing system. After the gas-sensing test, we opened the chamber and blew the test gas away with air. The response of the device was measured with a digital multimeter (Keithley 2000, Tektronix, Cleveland, OH, USA) and the data were read through LabVIEW on a laptop. Keithley 2000 implements resistance measurement by applying a constant current to the device and sampling the voltage. All the sensors worked at room temperature throughout the measurement.

### 2.3. Characterization Techniques

Field emission scanning electron microscopy (FESEM, GeminiSEM 500, Zeiss, Jena, Germany) was used to investigate the morphology of prepared samples. X-ray photoelectron spectroscopy (XPS, ESCALAB Xi+, Thermo Fisher Scientific, Waltham, MA, USA) analysis was performed to investigate the elemental composition of the samples of polyaniline, GO, and rGO. ESCALAB Xi+ has an Al/Mg double anode target X-ray source. When the energy scanning range is 0–1500 eV, the minimum scanning step size is 6 meV for the elemental composition analysis. The analysis area of ESCALAB Xi+ is 20 μm–8 mm.

## 3. Results

### 3.1. Material Characteristics

**Figure 3:**
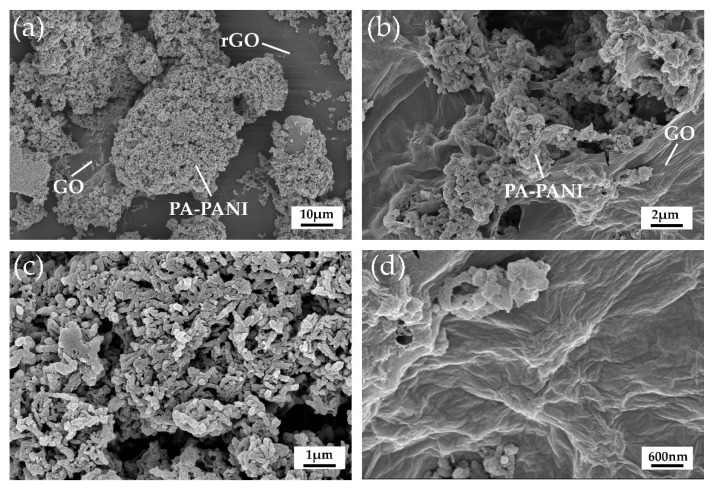
Characterization of materials by scanning electron microscopy. (**a**) The FE-SEM image of GO+PA-PANI/rGO composite sensitive membrane. (**b**) The FE-SEM image of PA-PANI and GO mixture. (**c**) The FE-SEM image of PA-PANI from (**a**). (**d**) The FE-SEM image of GO from (**b**).

Field emission scanning electron microscopy (FE-SEM) was used for material characterization, and the results are displayed in Figure 3a–d. Figure 3a is the FE-SEM image of GO+PA-PANI/rGO composite sensitive membrane synthesized in the work for ammonia detection. The center of Figure 3a displays the cluster structure of PA-PANI and the irregular flocculent PA-PANI polymer with about 100 nm diameter could be seen when the image is enlarged as shown in Figure 3c. The background of Figure 3a shows rGO film which indicates good integrity and flatness. Most areas of rGO are very clean and flat, however, a small part has some wrinkles. Figure 3b indicates the good interaction property of GO and PA-PANI. Morphology of GO is displayed in Figure 3d, which is a magnification form of the lower right corner of Figure 3b.

**Figure 4:**
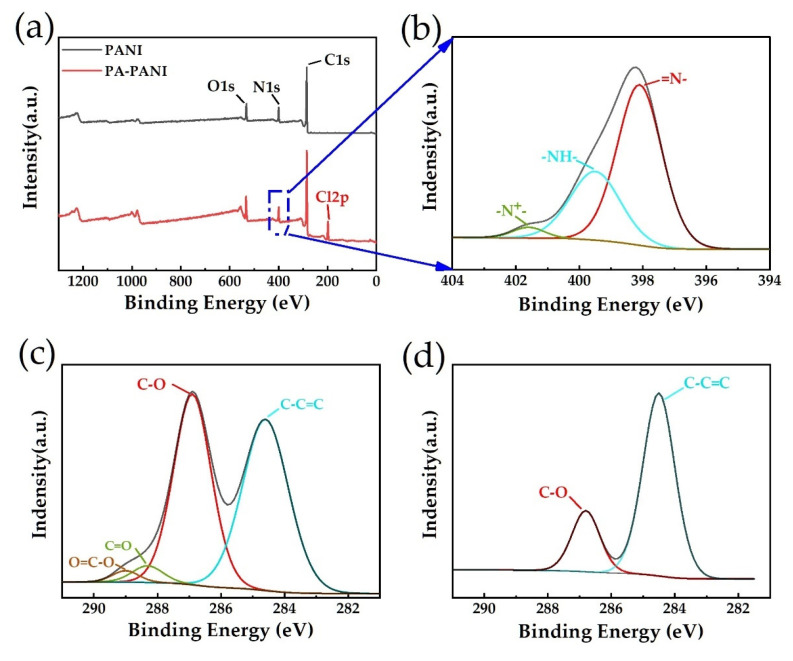
Characterization of materials by X-ray photoelectron spectroscopy. (**a**) XPS survey spectra of PA-PANI and PANI. (**b**) Zoom-in and fitting plot of PA-PANI’s N1s peak in XPS spectra. (**c**) Zoom-in plot and fittings of GO’s C1s peak in XPS spectra. (**d**) Zoom-in plot and fittings of rGO’s C1s peak in XPS spectra.

This experiment also carried out X-ray photoelectron spectroscopy (XPS) analysis to characterize the elemental composition of polyaniline before and after proton acid doping. Figure 4a illustrates the spectra of PANI and PA-PANI, which has C1s peak at 285 eV, N1s peak at 399.96 eV, O1s peak at 532.2 eV [31,32]. Compared to PANI, PA-PANI has 7.13% chlorine atomic additionally at 198.2 eV. The appearance of Cl 2p peak can significantly improve that PANI was successfully doped by hydrochloric acid. Besides, N1s peak of PA-PANI are analyzed to verify that plenty of N atoms are successfully protonated. The N1s peak of PA-PANI can be split into three peaks at the binding energy of 398.1, 399.4, and 401.6 eV [33], which correspond to =N-, -NH-, and -N+-, respectively. Among them, -N+- is the protonated part of N atoms which occupies 4.7%. In addition, XPS spectroscopy can also provide information about the element valence and bonding of GO and rGO. As p-electrons from the sp_2_ carbon determine the optical and electrical properties of graphene-related materials in most cases, the analysis of sp_2_ bonding can give us insight into the properties and structures of materials [34,35]. Thus, the C1s XPS spectrums of GO and rGO indicate the considerable content of oxidation groups on their surface. Therefore, in this work, to investigate detailed information of the chemical bonds in GO and rGO, the C1s peak at 285.72 eV are analyzed by AVANTAGE and the results are shown in Figure 4c,d. As shown in Table 1, C1s peaks of GO are split into four peaks at binding energy of 284.60, 286.90, 288.31, and 289 eV, which correspond to C-C=C, C-O, C=O, and O=C-O, and each of them occupies 49.72%, 45.14%, 3.24%, and 1.9% of the atomic content, respectively [36,37,38]. Nevertheless, for the atomic content in rGO, O=C-O and C=O disappear from the spectrogram completely while C–O has a sharp decrease to 22.51% and C-C=C increases to 77.49%. These results indicate the GO was partially reduced to rGO in L(+)- ascorbic acid solution. 

### 3.2. Gas-Sensing Experiment

The original resistance of the composite film with different PA-PANI/ GO’s mixing ratio ranges from 3.7 K–5.6 KΩ. The sensitivity test obtains the change in the resistance of the sensors. In order to deliver clear and visualized data to make comparisons, the corresponding relative responses of the sensors to NH_3_ are discussed in the following paragraphs. Further, the sensor’s response can be defined as Equation (1):

Response = [(R − R_0_)/R_0_] × 100%, (1)

where R_0_ is the original resistance of the composite sensitive membrane before injecting NH_3_ gas, and R is the real-time resistance of the composite sensitive membrane during the NH_3_ sensing experiment.

**Figure 5:**
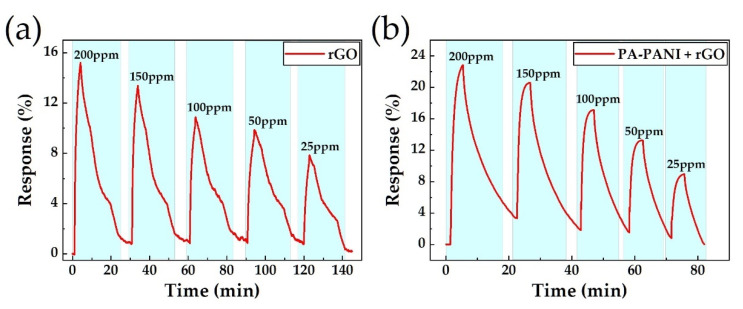
The relative NH_3_ gas-sensing test results. (**a**) Test results of pure rGO membrane-based sensors. (**b**) PA-PANI/rGO composite membrane-based sensors.

We firstly made up rGO and PA-PANI/ rGO composite membrane comparison groups to explore the phenomenon of ammonia sensing enhancement property with PA-PANI loading. As shown in Figure 5a, the sensitive membrane with only rGO reduced by L(+)- ascorbic acid solution merely gives response at 15.2% to 200 ppm ammonia, which mainly resulted from residual oxygen-containing groups on the surface of rGO that were substantiated by XPS spectra in Figure 4d. Nevertheless, rGO decorated with PA-PANI has a much more prominent response as shown in Figure 5b. Compared to pure rGO, rGO decorated with PA-PANI has a maximum enhancement of 50.2% to 200 ppm NH_3_. In previous literature reports, polyaniline doped by hydrochloric acid demonstrated p-type electrical conductivity [39]. At the same time, it is worth noting that NH_3_ is a kind of reducing gases, which tend to give electronics to other sensitive materials. Thus, consistent with the conclusions shown in Figure 5, PA-PANI is an ideal material for ammonia detection. Besides, when response time is accurately controlled to 3 min every testing cycle, it can clearly be obtained from the curves in Figure 5 that the sensor with only the rGO membrane does not appear to have any tendency to saturate. However, the response of the PA-PANI/ rGO composite membrane has already tended to saturate, and this phenomenon seems much obvious to 150, 100, 50, and 25 ppm NH_3_, especially. The result indicated the faster response to NH_3_ for the PA-PANI/ rGO composite membrane. Moreover, the recovery curve for the pure rGO membrane shows a lousy roughness characteristic, combined with a longer recovery time. By contract, the PA-PANI/ rGO composite membrane provides a rough recovery curve with low noise and a decline of recovery time at 48% compared to pure rGO on average.

**Figure 6:**
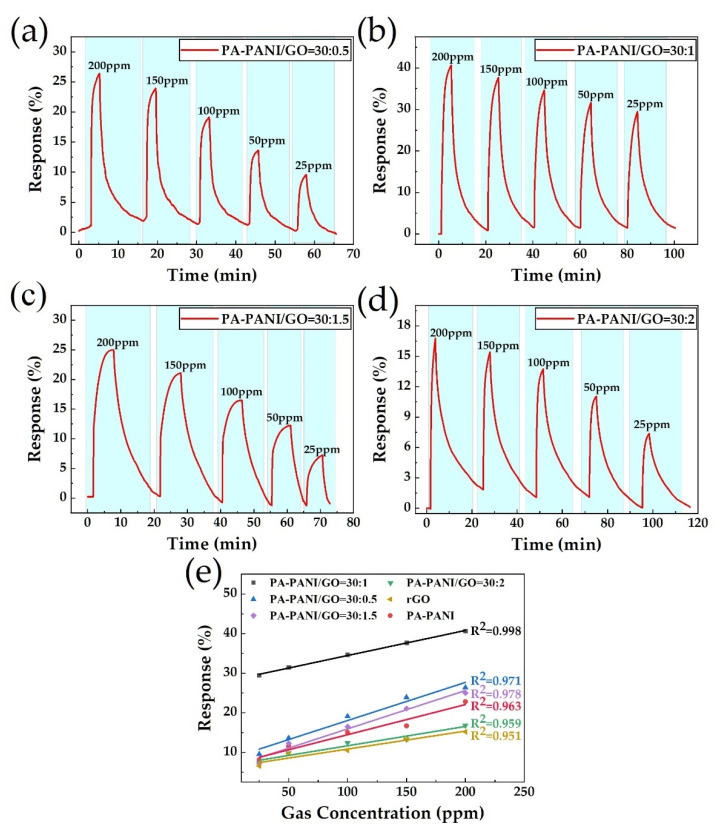
Gas-sensing response based on mixing ratio of (**a**) 30:1 PA-PANI and GO; (**b**) 30:1.5 PA-PANI and GO; (**c**) 30:0.5 PA-PANI and GO; (**d**) 30:2 PA-PANI and GO; (**e**) Linear regression of response for different mix proportions of every group.

Further, in order to improve gas sensitivity, we tend to make some decorations in PA-PANI. Some first-principles calculations have already proved that the appropriate introduction of defects and doping can enhance the sensitivity of graphene-based gas sensors [40,41]. Thus, GO seems to be a good choice, as it brings plenty of organic functionalizations to the surface and is quite easy for synthesizing in the laboratory. It is worth considering that carboxyl usually displays the character of hydrogen-bonding acidity, which both apt to incorporate with amido from PA-PANI and show advances in NH_3_ adsorption. Therefore, we mixed PA-PANI with GO to bring molecular adsorbed sites. In this work, we made different mixing ratios of PA-PANI and GO at 30:0.5, 30:1, 30:1.5, and 30:2. The results of gas-sensing response are displayed in Figure 6a–d and the linear fitting of gas-response for different mix proportions are shown in Figure 6e. 

As shown in the response curves displayed above, when mixing PA-PANI with a little bit of GO, the response is further improved. Especially to the situation of 30:1 mixing ratio, as shown in Figure 6a, the responsivity to 200 and 25 ppm NH_3_ reaches 40.56% and 29.46%, respectively. The optimal relative response improvement achieves 262.5% to 25 ppm and 78% to 200 ppm compared to the result of nothing mixed with PA-PANI shown in Figure 5b, which indicates that a little bit of GO doping in PA-PANI as decoration material would significantly enhance the NH_3_ gas-sensing behavior. The result is attributed to the functionalized groups, which provided molecular grippers and adsorption sites for NH_3_, on GO. Besides, from the linear fitting of gas-response for different decoration proportions, it is obvious that the group of 30:1 mixing ratio also has the best regression coefficient at 0.998. However, due to the lack of electrical conductivity, too much GO dose in the decoration material will block the possible charge transfer path and result in a degradation of the gas-sensing performance. 

### 3.3. Mechanism Analysis 

**Figure 7:**
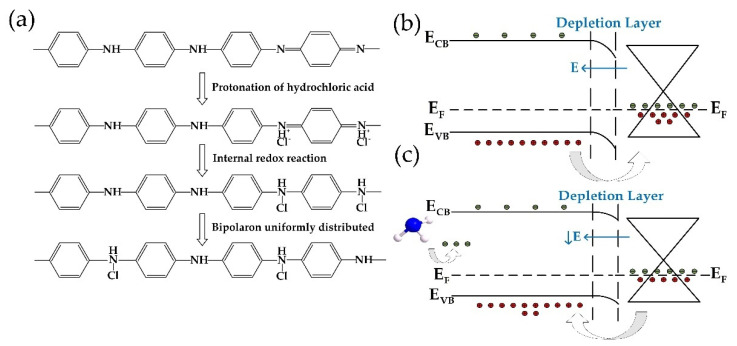
(**a**) Process of hydrochloric acid doping intrinsic PANI. (**b**) Schematic of the junction between PA-PANI and rGO. (**c**) Schematic of charge transport through the junction between PA-PANI and rGO when NH_3_ is adsorbed.

The chemical process of PA-PANI synthesizing and the possible mechanism for gas-sensing improvement are shown in Figure 7. When doped with protic acid, the number of electrons does not change. In the doping process, H^+^ firstly protonates the nitrogen atom on the imine, which makes the valence band of the doping segment on the polyaniline chain appear with holes, that is, P-type doping, forming a stable delocalized form of poly (emerald-imine) atomic group. The positive charge of the imine nitrogen atom is dispersed to adjacent atoms along the molecular chain through conjugation, which improves the stability of the entire molecular chain [42]. The effect of an external electric field, through the resonance of the conjugated electrons, causes the hole to move along the entire chain, showing electrical conductivity.

As rGO is primarily made up of honeycomb hexagonal rings of carbon atoms, while PA-PANI also has plenty of benzene rings on its molecular links, rGO and PA-PANI have some similarities in material structures. Based on semiconductor heterojunction theory, materials with similar crystal structures and atomic spacing can form a heterojunction at the contact interface. Because rGO was slightly reduced in the ascorbic acid (as indicated in Figure 3d) and polyaniline was heavily doped by hydrochloric acid, rGO synthesized in this work has fewer carriers and lower conductivity. Therefore, when PA-PANI and rGO contact, a hole diffuses from PA-PANI to rGO and leads to the formation of a depletion layer and a built-in electric field from rGO to PA-PANI as shown in Figure 7b. As a result, PA-PANI and rGO form a p-p^-^ junction. When the junction forms, as a kind of reducing gas, ammonia can be considered as a donor when chemical adsorption occurs on the surface of the sensitive membrane, and the amount of gas adsorbed is mainly enhanced by GO doping. When NH_3_ provides electrons to the PA-PANI, electron concentration inside the PA-PANI increases. The electrons brought by NH_3_ reduce the doping level of PA-PANI, and further decrease the built-in electric field at the interaction of rGO and PA-PANI. Therefore, some of the hole in rGO would flow back to PA-PANI, which decreases the hole concentration in rGO, as indicated in Figure 7c. The final result of the carrier concentration decreasing inside rGO is consistent with the gas-sensitive response data of resistance increase. Note that due to its chain rigidity and strong chain interaction, PA-PANI has poor solubility and is almost insoluble in most common organic solvents, even in strongly polar liquid like DMF. Moreover, the amount of PA-PANI dripping on the rGO surface is tightly controlled during sensor fabrication. So, the morphology of PA-PANI decorated on rGO’s surface demonstrates the character of clusters instead of the continuous thin film. Thus, the resistance of the composite membrane is mainly provided by rGO which is directly connected to IDEs. Based on the mechanism mentioned above, the resistance of the composite membrane would increase during gas detecting, which is consistent with the experimental results.

## 4. Conclusions

In conclusion, a composite sensitive membrane of a PA-PANI/GO mixture decorated rGO was synthesized and tested as a gas-sensing material. When the mixing ratio of PANI and GO is 30:1, the highest improvement of NH_3_ gas-sensing response achieves 262.5% at 25 ppm NH_3_ compared to no GO decorating in PA-PANI. The improved gas-sensing performance can be explained as GO provides effective NH_3_ adsorption sites while PA-PANI is responsible for the charge transmission. A reasonable charge transmission process is proposed: NH_3_ brings N-type doping to PA-PANI and leads to hole transport from rGO to PA-PANI, which finally induces the gas-sensitive response. Such a gas-sensing mechanism enriches the strategy of composite gas-sensing material design.

## Figures and Tables

**Table 1:** XPS analysis of C1s peak of GO and rGO.

Bands	GO	rGO
C-C=C (284.60 eV)	49.72%	77.49%
C-O (286.90 eV)	45.14%	22.51%
C=O (288.31 eV)	3.24%	none
O=C-O (289 eV)	1.9%	none

## Data Availability

Not applicable.

## Supplementary Materials

The following are available online at https://www.mdpi.com/article/10.3390/ma14112829/s1, Figure S1: The figures for polyaniline before and after protic acid treating, Figure S2: The response curve of the repeatability test for membrane with the mixture ratio of 30:1 in 25 ppm NH_3_.
