# Supplementary material for: Improving Ammonia Detecting Performance of Polyaniline Decorated rGO Composite Membrane with GO Doping
Source: Materials (Basel). 2021 May 25;14(11):2829. doi: 10.3390/ma14112829 (PMC8198450; doi:10.3390/ma14112829)
Supplement: Supplementary file 1 [file materials-14-02829-s001.zip › materials-1200952-supplementary.pdf]

Supplementary Information

Improving Ammonia Detecting Performance of Polyaniline Decorated rGO Composite Membrane with GO Doping

Yubin Yuan[1], Xiangrui Bu[1], Haiyang Wu[1], Qiang Wu[1], Xuming Wang[1], Chuanyu Han[1], Xin Li[1,2], Xiaoli Wang[1,3] and Weihua Liu[1,4,*]

1 School of Microelectronics, School of Electronics and Information Engineering, Xi'an Jiaotong University; yuan8262xy@stu.xjtu.edu.cn (Y.Y.); bxr1212@stu.xjtu.edu.cn (X.B.); wuhaiyang@stu.xjtu.edu.cn (H.W.); w18801757@163.com (Q.W.); hanchuanyu@mail.xjtu.edu.cn (C.H.); lx@mail.xjtu.edu.cn (X.L.); xlwang@mail.xjtu.edu.cn (X.W.)

2 Guangdong Shunde Xi'an Jiaotong University Academy, NO.3 Deshengdong Road, Daliang, Shunde District, Foshan 528300, China

3 School of Science, Xi'an Jiaotong University, Xi'an 710049, China;

4 Research institute of Xi'an Jiaotong University (Zhejiang), Hangzhou, Zhejiang 311215, China

* Correspondence: lwhua@mail.xjtu.edu.cn; Tel.: +86-29-8266-3343

Photograph from the experiment

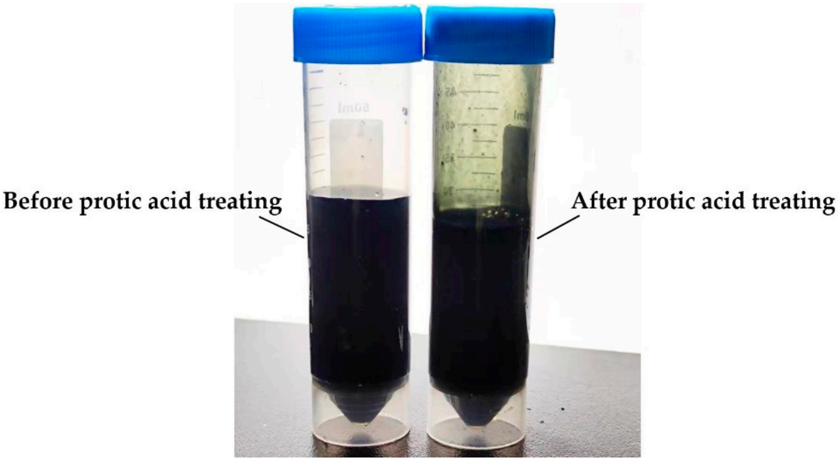

Figure S1. The figures for polyaniline before and after protic acid treating.

Result of repeatability testing

In the experiment, we performed a repeatability test of 25 ppm ammonia on the sample of 30:1 mixture ratio. Figure S2 indicates the result of the repeatability test. After four repeated tests, the device shows good repeatability and stability.

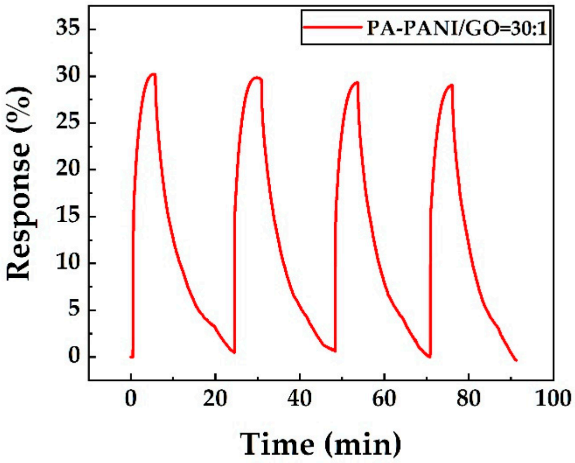

Figure S2. The response curve of the repeatability test for membrane with the mixture ratio of 30:1 in 25 ppm NH_3.
